# Skeletal Muscle Transcriptome Alterations Related to Declining Physical Function in Older Mice

**DOI:** 10.3390/jal3020013

**Published:** 2022-05-31

**Authors:** Ted G. Graber, Rosario Maroto, Jill K. Thompson, Steven G. Widen, Zhaohui Man, Megan L. Pajski, Blake B. Rasmussen

**Affiliations:** 1 Department of Physical Therapy, East Carolina University, Greenville, NC 27834, USA; 2 Department of Biochemistry & Molecular Biology, University of Texas Medical Branch, Galveston, TX 77555, USA; 3 Bioinformatics and Analytics Research Collaborative, University of North Carolina-Chapel Hill, Chapel Hill, NC 27514, USA

**Keywords:** sarcopenia, frailty, physical function, gene expression, RNAseq

## Abstract

One inevitable consequence of aging is the gradual deterioration of physical function and exercise capacity, driven in part by the adverse effect of age on muscle tissue. We hypothesized that relationships exist between age-related differentially expressed genes (DEGs) in skeletal muscle and age-associated declines in physical function and exercise capacity. Previously, male C57BL/6mice (6m, months old, 24m, and 28m) were tested for physical function using a composite scoring system (comprehensive functional assessment battery, CFAB) comprised of five well-validated tests of physical function. In this study, total RNA was isolated from tibialis anterior samples (n = 8) randomly selected from each age group in the parent study. Using Next Generation Sequencing RNAseq to determine DEGs during aging (6m vs. 28m, and 6m vs. 24m), we found a greater than five-fold increase in DEGs in 28m compared to the 24m. Furthermore, regression of the normalized expression of each DEG with the CFAB score of the corresponding mouse revealed many more DEGs strongly associated (R ≥ |0.70|) with functional status in the older mice. Gene ontology results indicate highly enriched axon guidance and acetyl choline receptor gene sets, suggesting that denervation/reinnervation flux might potentially play a critical role in functional decline. We conclude that specific age-related DEG patterns are associated with declines in physical function, and the data suggest accelerated aging occurring between 24 and 28 months.

## 1. Introduction

Aging-related decline of physical function is accompanied with, or predicated by, loss of skeletal muscle mass and strength (sarcopenia). Declining physical function and muscle health leads to reduced ability to perform activities of daily living, lower quality of life, development of disability, loss of independence, and increased mortality [1–3]. Sarcopenia is likely multifactorial with a varied etiology including disuse atrophy, neuromotor deficits, denervation, muscle quality reduction, and alterations to key proteins and cell signalling pathways [4–8]. There is a wide range of individual variability in the rate of declining physical and contractile function [9–12]. For this study, we hypothesized that changes in gene expression during aging would be highly correlated with functional loss. The primary goal was to determine which age-related changes to skeletal muscle gene expression are related to physical function and exercise capacity. The long-term goal is to identify potential mechanisms that might identify novel therapeutic targets.

Previously, we measured physical function and exercise capacity in 6-month-old (6m), 24-month-old (24m), and 28-month-old (28m) male C57BL/6mice, using our Comprehensive Functional Assessment Battery (CFAB), a composite scoring system comprised of five different well-validated determinants [rotarod (overall motor function), grip meter (forelimb strength), inverted cling (full-body strength/endurance), voluntary wheel running (volitional exercise and activity rates), and treadmill running (aerobic exercise capacity and endurance)] [9,10,12–14]. To further this work, we used Next Generation Sequencing (NGS) RNAseq to determine gene expression in total RNA extracted from the tibialis anterior (TA) muscles from a random subset of each age group from the original study [9]. Using linear regression, we determined strong associations of genes that changed expression with aging versus physical function (measured with CFAB), and identified numerous genes in muscle that may play a critical role in declining physical ability.

Studies of age-related changes in skeletal muscle gene expression have been well-covered in the literature [15–19]. In our current study, however, we combined our comprehensive physical function assessment with age-related gene expression data, revealing pertinent and novel information about the relationship between numerous genes and their potential role in functional aging.

We found thousands of genes changing expression (padj < 0.05, multiple comparisons adjusted *p* value, pval,) between 6m and 28m of age (6m–28m), but only a couple hundred between 6m and 24m (6m–24m). Likewise, we found hundreds of genes changing expression with age that also had strong (R ≥ 0.7) associations with CFAB in 6m–28m, but far fewer in 6m–24m. This discrepancy highlights the potential acceleration of biological aging over these four months, which is also expressed in many indicators of physical function and muscle health [9–14]. We used GOrilla (Gene Ontology enRIchment anaLysis and visuaLizAtion tool) and GSEA (Gene Set Enrichment Analysis) to determine highly enriched molecular function gene ontologies, which included cation transporters, and calcium transporters [20,21]. Overall, these findings establish a framework for understanding how aging alters skeletal muscle gene expression and how these gene expression changes are potentially linked to the gradual, inevitable, and progressive loss of physical function associated with aging, sarcopenia, and frailty.

## 2. Methodology

### 2.1. Mice

Three ages of C57BL/6male mice were obtained from the National Institutes of Health National Institute on Aging Charles River Aging Rodent Colony (a subset of mice from a previous publication [9] were randomly selected for this study: n = 8 for all at 6m, 24m, and 28m). Random selection was accomplished using a random number generator and samples were coded to ensure blinding until after RNAseq data were analyzed. The biostatistician was blinded to the groups. Mice were group-housed at 22 °C with a 12-h:12-h light/dark cycle, and food and water provided ad libitum. The characteristics of the mice are presented in Table 1.

Euthanasia was conducted under American Veterinary Medical Association (AVMA) Guidelines for the Euthanasia of Animals (2020). Mice were treated humanely using protocols approved by the East Carolina University and University of Texas Medical Branch IACUC committees, and conducted under appropriate and relevant guidelines and regulations. Methods were reported following ARRIVE guidelines (https://arriveguidelines.org, accessed on 5 May 2022) for the reporting of animal experiments.

### 2.2. Functional Testing

As previously published [9], CFAB is a composite scoring system that is composed of the following highly validated non-colinear determinants of physical function and exercise capacity: treadmill running (max speed test for endurance and aerobic exercise capacity), voluntary wheel running (VWR, for volitional exercise and activity rate), grip meter (forelimb strength), inverted cling (four-limb, total body strength and endurance), and rotarod (overall motor function, including balance, coordination, gait speed, endurance, and power production) [9,10,12–14]. Each mouse was rated for function by standardizing the results from each determinant to the mean and standard deviation (SD) of the reference group (6m) to create a score. Then we summed the individual determinant scores to create the CFAB score. For example, if the rotarod time for an individual mouse was 160 s, and the reference mean = 123.9 s and SD = 28.9, then the rotarod score for the mouse = 1.249 (the standardized value of the number of SD away from the reference mean). This same process was then performed for each determinant, and the resulting scores were added to produce the CFAB score of the mouse. Because the reference group were 6m mice, most older mice will have negative CFAB scores, indicating that those mice performed worse than the average adult mouse. A more negative CFAB is indicative of worse overall physical function. See the Supplemental Methods Section for more details on the specific tests.

### 2.3. Tissue Collection and Handling

As described previously [9], the mice were deeply anesthetized with ketamine/xylazine mix and euthanized via exsanguination and removal of the heart after non-survival surgery to collect the hindlimb muscles at the completion of the testing protocols. The muscles were blotted dry, weighed, and then immediately flash frozen in liquid nitrogen. Subsequently, the muscles were stored at −80 °C until total RNA extraction.

Total RNA extraction has been previously described [14,22]. In brief, we used Tri-Reagent (Molecular Research, #TR118) following the manufacturer’s instructions to extract total RNA from TA muscle, using the entire TA muscle. We quantified the extraction using a Nanodrop2000 (ThermoScientific, Waltham, MA, USA), with mean concentration 330.8 ± 24.3 ng/μL, 260/280 ratio 1.69 ± 0.020, 260/230 ratio 1.98 ± 0.09. We determined RNA integrity using an Agilent Bioanalyzer 2100; mean RIN was 9.23 ± 0.139. Two of the 24 total isolated RNA samples (n = 1 each from 6m and 24m groups) did not meet the standard lower limits for purity and integrity, and were not used for RNAseq.

### 2.4. NGS RNAseq

RNA samples (n = 22 total; n = 7 6m, n = 7 24m, and n = 8 28m) were quantified using a Qubit fluorometer and qualities were assessed with an Agilent Bioanalyzer. Poly-A + RNA was enriched from ~0.5 ug of total RNA and used as a template to generate sequencing libraries using the New England Biolab NEBNext Ultra II RNA Library Prep Kit following the supplier’s protocol. Libraries were pooled and sequenced on an Illumina NextSeq 550 High-output flow cell with the 75 base pair single-end protocol with two runs: 6m vs. 24m and 6m vs. 28m. The 6m samples were the same in both runs to serve as a control to facilitate comparison of the 24m and 28m groups. Libraries for the 6-month samples were prepared twice, once each in parallel with the 24m and 28m samples to help control for batch effects. Read counts from these technical replicates were kept separate in the analyses. The average number of reads per sample was 39,366,241 in the first run of 6m versus 24m and 35,287,066 for the second run of the 6m versus 28m. Greater than 93% of base calls had Illumina Q-scores above 30, and average Q-scores were above 34 for all samples. Raw NGS data is stored at the GEO record GSE152133.

## 3. Data Analysis

### 3.1. General

We used SPSS v24, v27, and v28 (IBM) to analyze the statistics. Data were reported as means ± standard error, unless otherwise designated. Significance was designated as *p* < 0.05, with trends reported if 0.05 < *p* < 0.10. CFAB, TA muscle mass, and the CFAB determinants used one-way ANOVA to detect differences in means. The individual test used is described in the text of the results. Post hoc analysis for ANOVA used least significant differences (LSD). The original data for CFAB and other measurements met assumptions of normality other than the inverted cling test which was transformed by log10 (see original study [9] for kurtosis and skew reporting). The log10 transformation version of the inverted cling is thus used to calculate CFAB [9].

#### RNAseq:

The reads were demultiplexed and aligned to the mouse mm10 genome using the splicing aware software STAR, version 2.5.4b, with the ENCODE recommended parameters [23]. The genome index was built with the Illumina iGenomes UCSC mm10 genomic sequence and annotation file, and reads mapping to genes were quantified with the STAR–quantMode GeneCounts option [23].

The read counts per gene for each sample were input into the DESeq2 differential expression program, version 1.22.2. [24]. Following the DESeq2 vignette, differentially expressed genes were called with an adjusted *p*-value cut-off of less than 0.05 and a log2 fold-change (log2fc) of ≥1.0 or ≤−1.0. We used the rlog function in DESeq2 to generate a table of log2 normalized counts, which we then used to generate the PCA plots and heatmaps. We used DESeq2 to create the PCA figures and the heatmap package in R to create the heatmaps [25]. The principal components analysis determined the gene sets that contributed most to the variability between the different age groups and identified which genes contributed most to explaining CFAB variation.

### 3.2. Further Data Analysis of RNAseq Data and CFAB Data

The Bioinformatics and Analytics Research Collaborative (BARC) at the University of North Carolina at Chapel Hill performed the following data analysis as consultants to the project. The code and datasets associated with these analyses are available in GitHub at https://github.com/MANZHAOHUI/Graber (established on 10 May 2023).

Linear regression between the log2 normalized gene counts of each differentially expressed genes (DEGs, independent variable) from each sample with the CFAB value, CFAB determinants, and TA mass (dependent variables) of the corresponding individual mouse/sample was used to evaluate the DEGs’ correlation coefficient (R) with the physical function measurements (CFAB score) of the mice from various ages. Python (v3.7.8, function: stats.linregress(x,y) from the python function ‘stats’ imported from the python package ‘scipy’) was used to generate metrics including ‘slope, intercept, r, *p*_value, std_err’. The ‘pvalue’ generated by ‘scipy.stats.linregress’, which uses Wald Test with t-distribution of the test statistic for a hypothesis test whose null hypothesis is that the slope is zero, was employed to indicate the correlation between the value of a given gene from a particular sample and corresponding CFAB value (from: https://docs.scipy.org/doc/scipy/reference/generated/scipy.stats.linregress.html, accessed on 23 July 2020).

GSEA (Gene Set Enrichment Analysis) was conducted using R, referring to the method explained in https://stephenturner.github.io/deseq-to-fgsea/ (accessed on 23 July 2020) against NGS datasets [21]. The reference database used was ‘MousePath_GO_gmt.gmt’ downloaded from http://ge-lab.org/gskb/ (accessed on 23 July 2020). Based on the results of GSEA, genes from the NGS datasets with the cut-off (|log2fc| ≥ 1, padj < 0.05) were further filtered into enriched gene sets that were significant, as indicated by a false discovery rate (FDR) of less than 25% (http://www.gsea-msigdb.org/gsea/doc/GSEAUserGuideTEXT.htm#_GSEA_Statistics, accessed on 23 July 2020). After this filtering, 127 and 1049 genes were left from 6m vs. 24m comparison and 6m vs. 28m comparison, respectively.

Next, Gene Ontology enRIchment analysis (GOrilla analysis) was rendered on http://cbl-gorilla.cs.technion.ac.il/ (accessed on 23 July 2020) against the selected DEGs from the two runs with the DEGs cut-off [20]. Enrichment is the over or under representation of differentially expressed genes in functional categories (the GOs/gene ontologies).

The final step was to intercept the results from both GSEA and GOrilla [20,21]. For the same input, GSEA and GOrilla generated different sets of enriched genes. We intersected the two gene sets and found the genes in common for both models. The intersection provides high confidence between two approaches for ascribing functional categories to the data. GSEA and GOrilla have similar purposes, but use different methods. GOrilla is the older tool, more traditionally used, and focuses on significant genes, whereas GSEA considers all of the genes in an experiment, not just those above an arbitrary cut-off in terms of fold-change or significance. Moreover, GSEA assesses the significance by permuting the class labels, which preserves gene-gene correlations and thus provides a more accurate null model.

### 3.3. Transcription Factor Analyses

After inputting the DEGs from Table S10 (|R| ≥ 0.70) into https://maayanlab.cloud/Enrichr/ (accessed on 20 April 2023), we report the ChEA 2022 results to analyze transcription (all terms with padj < 0.05) as a table in the Online Supplemental Section [26–29]. In addition, we also input the same dataset to Enrichr, and report results from Reactome and GO molecular function in the Supplement.

## 4. Results

### 4.1. CFAB

**Figure 1.**
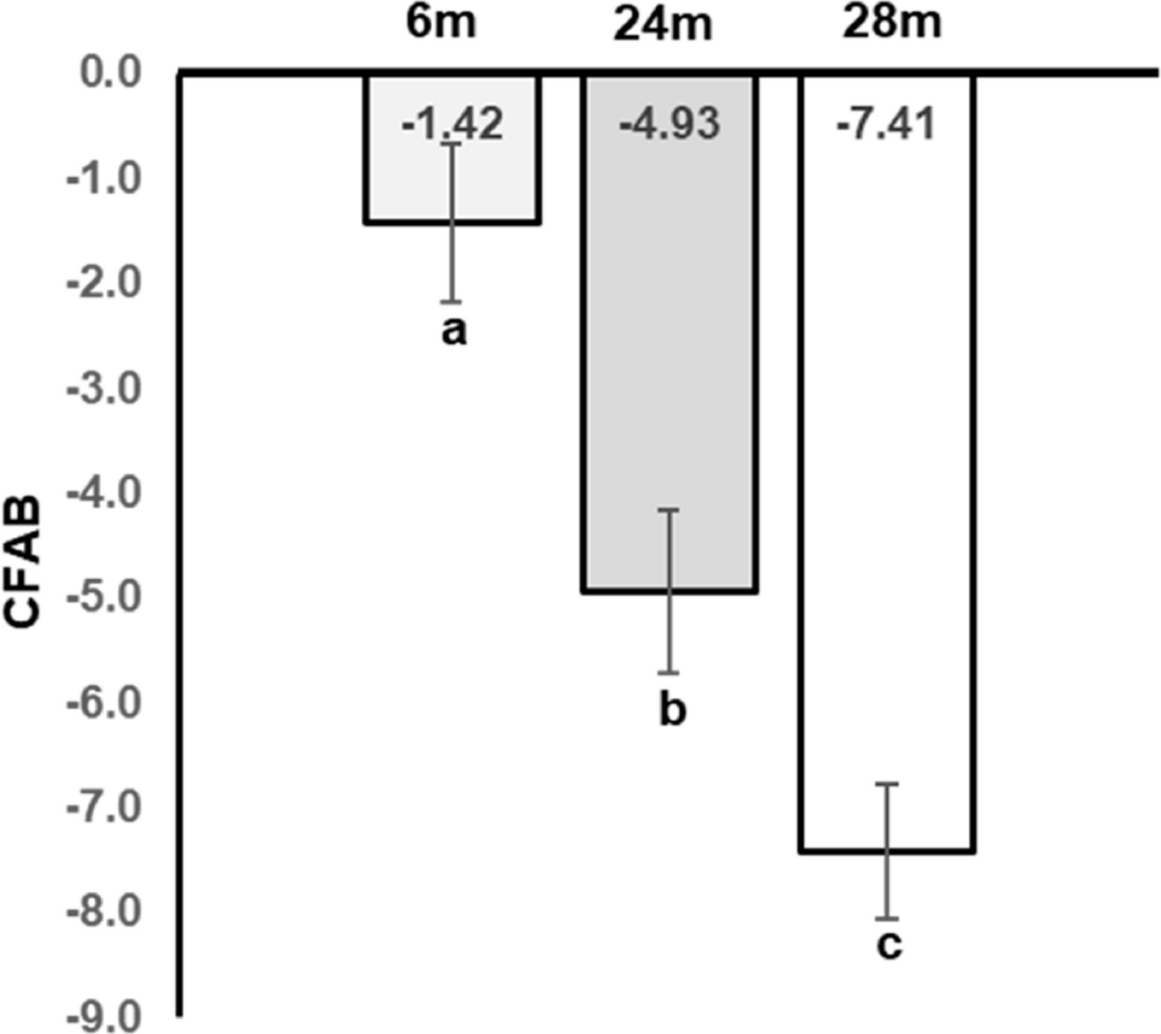
Declining Physical Function with Age. M = months, different letters indicate statistical significance. Statistics are from 1-way ANOVA with Least Significant Differences post hoc testing. Note: This data was a subset of previously published data and is shown for representative purposes only. For an understanding of the statistical relationships of the data, please refer to the original text [9].

The mice in this study demonstrated overall declining physical function with age, as measured with the CFAB component tests of rotarod, grip strength meter, inverted cling, treadmill, and voluntary wheel running. The CFAB score was significantly different between groups (*p* < 0.05) (see Figure 1). Since these mice were randomly selected from a larger cohort (e.g., n = 7 6m in the current work versus n = 30 6m in the parent study), the statistical results presented herein do not exactly match the results from the parent study, though trends are comparable. For a more complete discussion of the functional testing methods and results, consult our previously published work [9]. Standardized scores of the individual determinants are presented in the Online Supplemental Data Table S1.

#### 4.1.1. NGS RNAseq: (See the Full Raw Dataset on GEO at GSE152133)

##### Age-Related DEGs: 28m Compared to 6m

**Figure 2.**
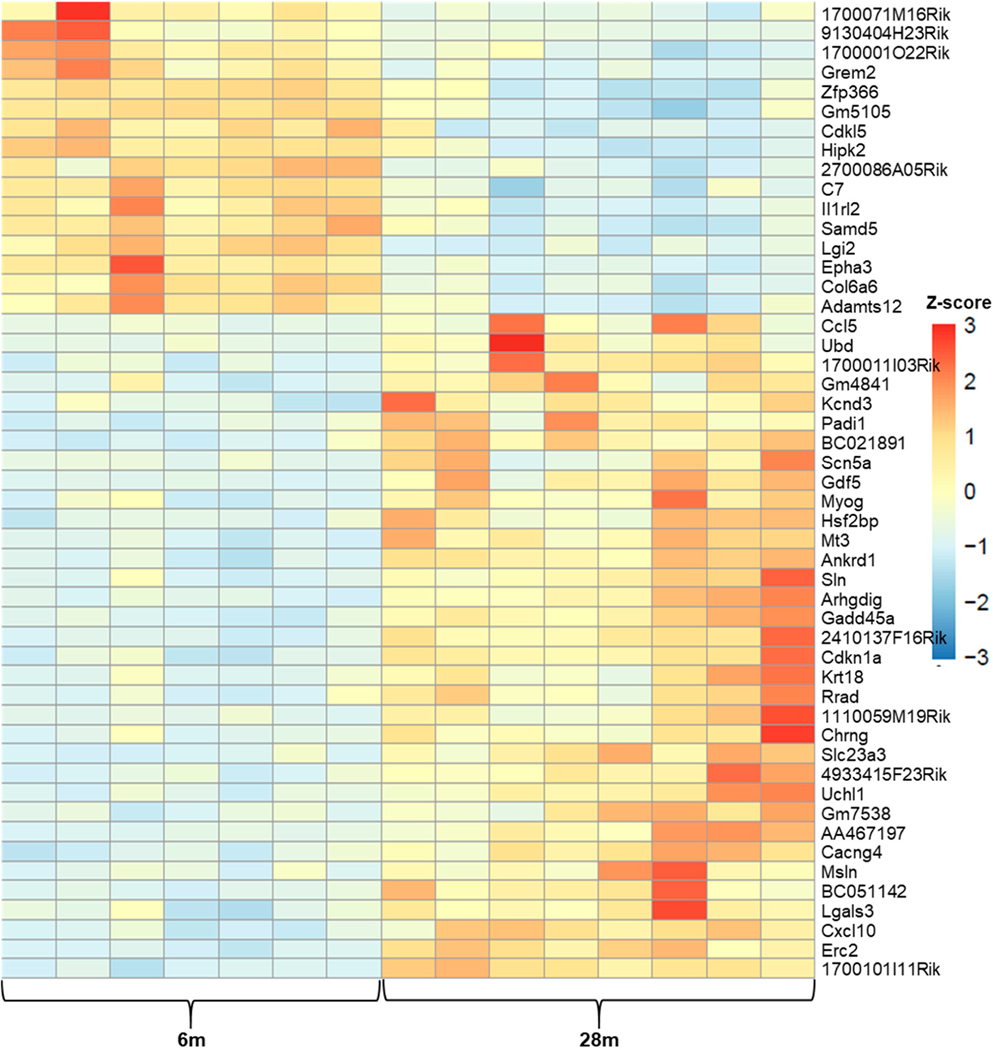
6m vs. 28m Top 50 Z-scores Heatmap. The names of the genes are to the right of each row, and each column = expression data from an individual mouse, 6m = 6-month-old and 28m = 28-month-old mice; color coded key to fold change z-score is on the right, with red the highest (+3) and dark blue the lowest (−3).

**Figure 3.**
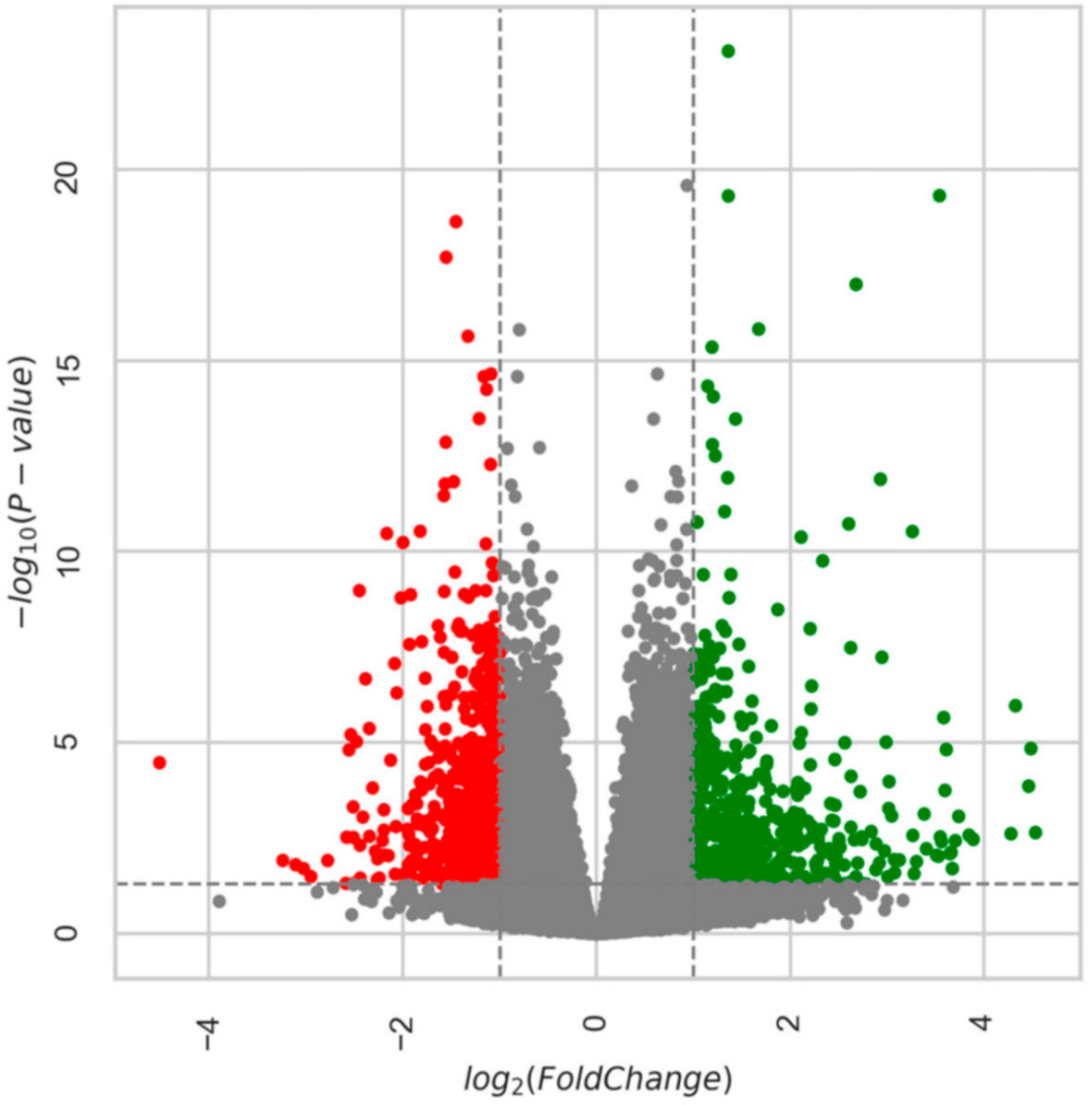
Volcano Plot: 6m vs. 28m. Each dot (red indicates downregulated gene expression with age and green indicates upregulated) represents one gene with the log2 fold change on the x-axis and the adjusted *p*-value on the y-axis. Dashed lines indicate the cut-offs of adjusted *p*-value < 0.05 (horizontal line) and log2 fold change >|1| as the two vertical lines. All colored circles were considered significantly different gene expressions with age. 6m = 6-month-old mice and 28m = 28-month-old mice.

**Figure 4.**
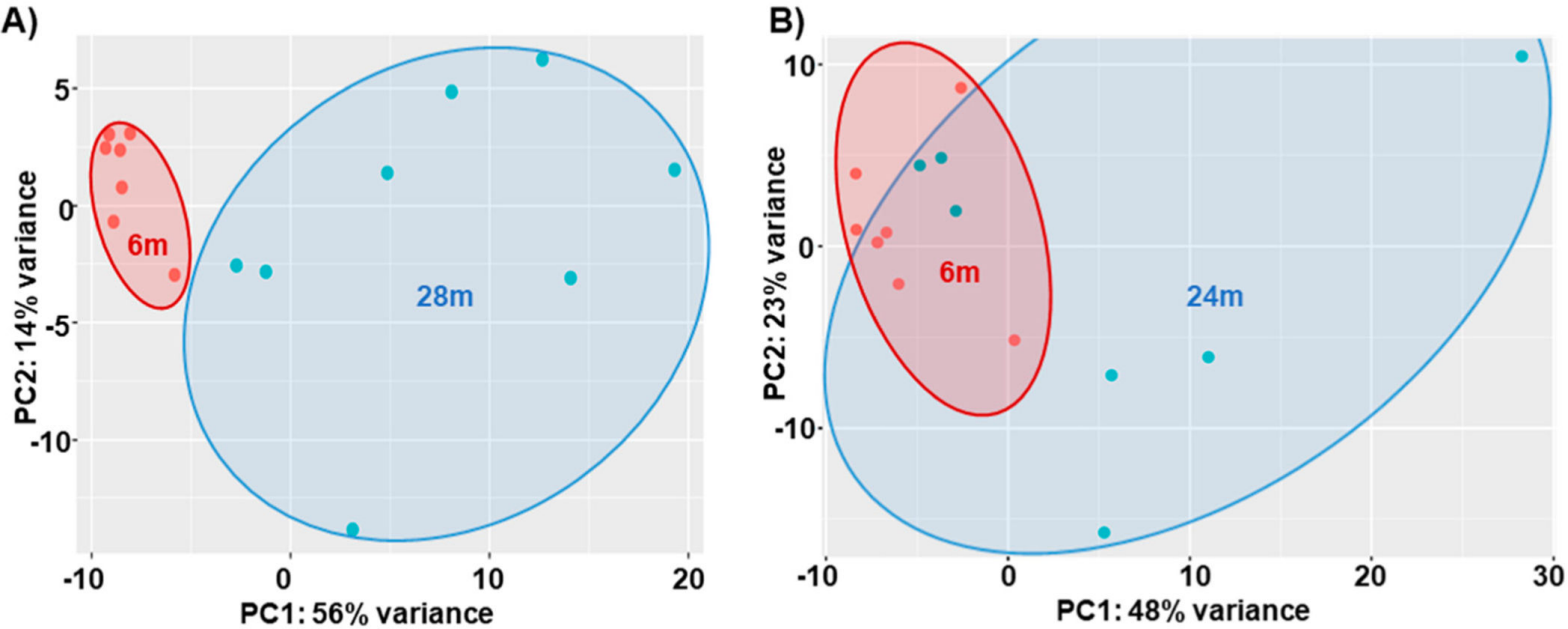
PCA Plots. (**A**) 6m vs. 28m; (**B**) 6m vs. 24m Key: 6m = 6 months old, 28m = 28 months old, PCA = principal components analysis, PC = principal component, and percent variance indicates how much of the variability between subjects is explained by the components, red dots = 6m and green dots = 28m.

Overall, in the 6m–28m data, there were 6707 genes with padj ≤ 0.05, and of these, 3153 were downregulated and 3554 were upregulated. After applying the a priori cut-off for DEGs (|log2fc| > 1, padj ≤ 0.05), there were 612 downregulated genes and 605 upregulated genes (total genes = 1217). Figure 2 is a heatmap showing the top 50 gene expression changes (by z-score) between 6m and 28m. Figure 3 is a volcano plot showing separation of the gene sets. Table 2 lists the top 20 genes upregulated with age (by log2fc), and Table 3 lists the top 20 downregulated genes (see Table S2 for all DEGs). In Figure 4A, the 2D principal component analysis (PCA) scores plot indicates a separation between 28m and 6m clusters, with no overlap. This result was confirmed by using the supervised multivariate analysis based on a partial least squares-discriminate analysis (PLS-DA) (component 1 [6m] was 14% and component 2 [28m] was 56%). Interestingly, the 28m gene expression is widespread in Figure 4A, indicating heterogeneity. There are groupings both closer to and farther away from the 6m, with a potential outlier as well.

We used GSEA and GOrilla to examine gene ontology enrichment in the 6m–28m DEGs (see Figure S1 and Tables S4 and S5 in the Supplement). After analyzing the RNAseq data with GSEA, there were 1049 validated DEGs remaining. Using GOrilla to further determine gene set enrichment in this comparison (using the same gene set as GSEA), there were 73 gene ontology terms enriched (minimum False Discovery Rate q-value, FDR q-val < 0.10; 72 terms FDR q-val < 0.05), ranging from a high enrichment of 26.29 to a low of 1.12. In all, there were 8 gene sets highly enriched, E, (E > 10, averaging 17.6 ± 6.0 sd, standard deviation), including: GO:0016907 (G protein-coupled acetylcholine receptor activity, enrichment, E = 26.3), GO:0098639 (collagen binding involved in cell-matrix adhesion, E = 25.7), GO:0048407 (platelet-derived growth factor binding, E = 21.9), GO:0008046 (axon guidance receptor activity, E = 14.3), GO:0035373 (chondroitin sulfate proteoglycan binding, E = 14.2), GO:0015464 (acetylcholine receptor activity, E = 13.6), GO:0005021 (vascular endothelial growth factor-activated receptor activity, E = 12.9), and GO:0030020 (extracellular matrix structural constituent conferring tensile strength, E = 12.0). The intersection of GOrilla and GSEA (Table S8) identified 359 DEGs.

##### Age-Related DEGs: 24m Compared to 6m Mice

There were fewer changes in gene expression in the 6m–24m than in the 6m–28m. There were 234 genes with padj ≤ 0.05; of those, 50 were downregulated and 184 were upregulated. There were 138 total DEGS with 130 upregulated and 8 downregulated (see Table S3 for the listing of all DEGs). In Figure 4B, the 2D principal component analysis (PCA) scores plot indicates an incomplete separation between 24m and 6m clusters, with evident overlap. Greater variance was observed in the 24m vs. the more homogeneous 6m clusters. This result was confirmed by using the supervised multivariate analysis based on a partial least squares-discriminate analysis (PLS-DA) (component 1, 6m, was 23% and component 2, 28m, was 48%).

We used GSEA and GOrilla to examine gene ontology enrichment in the 6m–24m DEGs (see Figure S2, Tables S3, S6 and S7 in the Supplement for further details). Analyzing the RNAseq data with GSEA, we found that 127 genes remained of those that fell under the DEG cut-off. Using the same genes fed to GSEA, GOrilla determined there were 19 gene ontology terms enriched (FDR q-value < 0.10; 18 terms FDR q-value < 0.05), ranging from a high enrichment of 43.19 to a low of 1.39. In all, there were 3 gene sets highly enriched (E > 10, averaging 32.5 ± 11.8 sd), including: GO:0001602 (pancreatic polypeptide receptor activity, E = 43.2), GO:0001601 (peptide YY receptor activity, E = 34.55), and GO:0004983 (neuropeptide Y receptor activity, E = 19.81). The intersection of GOrilla and GSEA identified only three genes for this 6m–24m comparison (Table S9).

### 4.2. Regressions of DEGs with CFAB

We primarily focused our attention on the changes that occurred in the transcriptome between the adults (6m) and the oldest group (28m). This was because the alterations in gene expression were most extreme at the advanced age (many more DEGs and many more DEGs strongly associated with CFAB), and we knew from previous work that the most profound changes in function, muscle health, and contractile ability occur at the older ages in mice [9,10,13]. See the datasets in the Online Supplemental Data Tables S10–S21 for more details.

Regression analysis of the 6m–28m determined there were 704 DEGs with at least a moderate (R ≥ |0.50|) correlation with physical ability (CFAB score); of these, 372 were strongly associated with CFAB (R ≥ |0.70|). Of these strongly associated genes, 239 were positively associated and 133 were negatively associated with CFAB. Of the genes that were positively associated, all were downregulated. Of the genes that were negatively associated, all were upregulated. Table 4 lists the top (by |R|) age-regulated genes associated with physical function in the 28m group (see Table S10 for all R > |0.70|).

The different determinants of CFAB (VWR, Grip, Inverted Cling, Rotarod, and Treadmill) in the 6m–28m were regressed in the same way as CFAB (See Tables S11–S15). Notably, out of the top 71 DEGs correlated with CFAB (|R| > 0.80), 35 of them are also in the top 30 DEGs (by |R|) of the determinants (see Table S16). However, 11 of the top 30 (by |R|) DEGs in the CFAB list do not appear in the top lists of the determinants. For example, the top DEG on the CFAB list, Dclk3 (Doublecortin like kinase 3, R = 0.899), was not in the top 30 in any of the determinant lists. Interestingly, VWR, Grip Test, and Inverted Cling contain the most top CFAB correlates. However, the VWR top DEG, Necab1 (N-terminal EF-hand calcium-binding proteins 1, R = 0.791), and the only VWR DEG with |R| > 0.70, was ranked at #237 on the CFAB list (R = 0.741).

In contrast, of the 6m–24m DEGs, regression analysis determined that there were only 55 genes with at least a moderate (R ≥ |0.50|) correlation with physical ability (CFAB score); of these, only 35 were strongly associated (all R < −0.70) with CFAB. Of the strongly associated genes, all 34 were negatively associated and downregulated (see Table S17 for details). The different determinants of CFAB (VWR, Grip, Inverted Cling, Rotarod, and Treadmill) were regressed with the age-associated DEGs in the 6m–24m group (See Tables S18–S22). Notably, only inverted cling and grip had any DEGs with |R| ≥ 0.70, 24 and 13, respectively (see Tables S19 and S20 for details).

In addition, we regressed the TA muscle mass (dependent variable) with the DEGS of both sets (for details see 6m–28m in Table S23 and 6m–24m in Table S24) and found that there were 595 DEGs with |R| ≥ 0.70 in the 6m–28m group, while there were only 3 in the 6m–24m group: Ak4 (R = −0.76, log2fc 1.48), Erc2 (R = −0.72, log2fc 3.41), and Car3 (R = 0.697, log2fc 1.05). Notably, in the 6m–28m analysis, all three were also on the list: Ak4 (28m, ranked #164, R = −0.839, log2fc 1.08), Erc2 (28m, #212, R = −0.824, log2fc 3.54), and Car3 (28m, #28, R = −0.900, log2fc 1.32).

There are far more DEGs at the older age (6m–28m) that might have a role in functional loss or are potentially involved in the regulation of muscle mass. More than 10-fold DEGs were identified to be strongly associated with function in the 6m–28m group (372) than the 6m–24m (35). Furthermore, different DEGs were among the top |R| values in the two groups, and there were different |R| values for the same DEGs in the two analyses, potentially indicating that some genes could play a more significant role in the decline of physical function depending upon the age of the mouse.

Furthermore, using the gene set from Table S10, we ran ChEA via Enrichr, and the resulting transcription factors (48 significant terms were returned) that overlap (padj < 0.05) with our DEGs (6m–28m, with |R| ≥ 0.70) are presented in Table S25. The transcription factors with the highest combined scores included WT1, MTF2, CEBPD, SUZ12, SOX9, TP53, YAP1, ZNF217, ESR1, and DROSHA (48.2, 26.5, 25.5, 24.5, 21.6, 21.2, 18.9, 18.9, 18.6, and 18.2, respectively). Interestingly, CEBPD is a transcription factor DEG that we found to have reduced expression with age (log2fc −1.025), and that is strongly associated with CFAB (R = −0.792).

## 5. Discussion

### 5.1. Physical Function Declines with Aging

It is well-established that both rodents and humans lose muscle mass and strength as they get older. Alongside this decline comes a decline in physical function and exercise capacity [8,9,11,12,29,30]. Reductions in power production and contractile velocity have been shown to precede loss of strength and mass, indicating that deterioration other than atrophy contributes to the onset of muscle dysfunction [1,11].

Various hypotheses have been proposed to explain mechanisms of both early onset loss of power and the disconnect between mass retention and strength loss in the context of declining physical performance. Loss of so-called “muscle quality” is one such theory. During aging, the infiltration of fat, connective tissue, and scar tissue into a muscle can reduce the overall cross-sectional area devoted to contractile units while altering structural parameters of the tissue, and combined with other macro level alterations such as tendon stiffening, may reduce power and strength at the whole muscle level [5,8,11,31,32]. Additionally, at the cellular level, many deleterious changes with aging can affect muscle contraction, such as post-translation modifications to key contractile proteins, fiber-type shift, enhanced preferential denervation of type 2 motor units, cell signaling abnormalities, autophagy dysregulation, and reduced mitophagy leading to enhanced reactive oxygen species (ROS) production [6,8,33–35].

In this study, we set out to obtain a mouse skeletal muscle transcriptome profile to directly compare to declining physical function. Our goal was then to develop mechanistic hypotheses based on this relationship. There were numerous associations between various DEGs and the overall state of functional health (measured by CFAB). Using GOrilla and GSEA analysis of gene ontology, we examined potential cellular processes in flux. Calcium handling, denervation, neuromuscular junction dysfunction, and motor neuron alterations were some implicated processes. See the Supplemental Sections for more details (Figures S1 and S2, Tables S2–S21). In the following sections, we distinguish our data with *italics* when discussing similarities and differences with other studies from the literature.

### 5.2. Age-Related Gene Expression Relationship with CFAB

We determined relationships with the data from linear regression of the CFAB score (generalized physical function as the dependent variable) versus differential gene expression (independent variable) at both the 6m–24m and 6m–28m ages. Our findings suggest that gene expression may change and affect function differentially based on age. One example is Erc2 (or Cast1), coding for ELKS-Rab6-interacting protein 2 (many roles, including organization of the cytoskeleton structures involved in pre-synaptic vesicle release) [36,37]. *Erc2* has a *log2fc 3.41* at 6m–24m, and *log2fc 3.54* at 6m–28m, a similar increase of expression at both ages. However, *Erc2* negatively correlates with CFAB at 6m–24m *R* = −*0.71* and at 6m–28m *R* = −*0.88*, potentially indicating a more robust relationship with physical function as the mice get older. While correlation does not equal causation, it is clear the association of Erc2 with CFAB increased in the older age group. Therefore, Erc2 may be more critically related to neuromuscular performance at advanced age (28m) than at 24m, when function is also better preserved.

### 5.3. Potential Mechanisms of Functional Age

We observed some common themes related to the protein function of some of the top gene changes (See Tables 1–3) such as calcium handling dysregulation (*Sln,* sarcolipin with *log2fc 4.33*), denervation (*Achg, acetyl choline receptor gamma, log2fc 3.599*) and neuromuscular junction degeneration, and proteolytic process regulation (*Ubd, ubiquidin log2fc 4.46*). See also the results of the gene ontology in Figures S1 and S2. In the next sections we discuss our findings related to transcription factors and denervation (*results from our current study are shown in parentheses*), followed by further comparisons of our findings to the existing literature. The Supplemental Discussion details gene expression changes we uncovered that are potentially related to disuse atrophy and calcium handling.

### 5.4. Transcription Factor Gene Expression with Aging

A recent study narrowly focused on multi-tissue conserved epigenetic regulation and the transcriptome (genes within 5000 base-pairs of transcription start sites) used the quadriceps of 6 and 24-month-old mice as part of their gene array analysis [38]. It is interesting to note that while they only reported and investigated a very narrow scope of genes, many of their top genes did not change significantly in our TA transcriptome within our cut-offs (adj. *p* < 0.05 and log2fc ≥ 1) at 6m and 24m—perhaps partly indicating a potential difference between the highly glycolytic fast twitch TA and the more oxidative quadriceps. Another likely explanation for the differences is that our DEG cut-off was much more stringent than the Sleiman et al. study where they included all genes with a false discovery rate of <10%, regardless of fold change.

However, if we instead compare 6m TA to 28m TA, we do see some evidence of transcriptional regulation alterations similar to Sleiman and colleagues [38]. By widening our scope of significance to include genes with padj < 0.05 and any log2fc, we see similar results in our data to what Sleiman and colleagues discovered: for transcription factors related to aging such as the SREBF family motifs (*SREB1 log2fc* −*0.61, and SREB2 log2fc* −*0.67, both padj* < 0.001) that are known to regulate lipid homeostasis, and *Mecp2* (*log2fc* −*0.30, padj* = 0.006) that represses expression of genes and is a regulator of normal neuron function. We also see significant age-associated expression changes of members of the Zbt family (*log2fc: Zbtb37* −*1.06, Zbtb7c* −*0.63, Zbtb46* −*0.73, Zbtb22 0.25, Zbtb48 0.31, Zbtb33* −*0.29, Zbtb10* −*0.33, Zbtb5* −*0.30, Zbtb11* −*0.22, and Zbt20 0.34*) that code for the zinc finger and BTB domain-containing proteins which are known as transcriptional repressors. For example, ZBTB20 promotes production of pro-inflammatory cytokines by downstream activation of Toll-like receptors. In support, we uncovered that *Nfkbia* (Nuclear factor of kappa light polypeptide gene enhancer in B-cells inhibitor, alpha) increased expression *by log2fc 0.96*, and thus could be involved in reducing NF-κB transcription factor (confirmed significant reduced expression of *Nfkb1* is *log2fc* −*0.45*). Therefore, upregulation of ZBTB20 in our older mice would support increased inflammation—one hallmark of aging and one of the likely mechanisms contributing to muscle atrophy.

Furthermore, we inputted our list of DEGs from Table S10 (|R| ≥ 0.70) into https://maayanlab.cloud/Enrichr/ (accessed on 18 March 2023) to analyze transcription [33–35]. Interestingly, the transcription factor *CEBPD* (CCAAT Enhancer Binding Protein Delta) with the third highest combined score *(25.543)* was also on the list of DEGs, being downregulated and negatively associated with CFAB (*log2fc* −*1.025, and R* = −*0.792*). CEBPD had 20/413 curated genes present in our dataset (*Usp244, Cdk19, Mbd5, Bmpr2, Nhlrc3, Mme, Crybg3, Lamc3, Heg1, Nbeal1, Sorcs1, Lamc1, Bicc1, Btaf1, Palld, Psd3, Rel, Fat1, Wdfy3, and Trpm3*). CEBPD is a tumor suppressor and an important regulator of immune and inflammatory response [39]. CEBPD may also be involved in cell motility by altering cytoskeletal dynamics [40]. The data suggests that CEBPD could potentially be a master transcription factor regulator of age-related gene expression related to physical function that is itself regulated with age. Further investigation is warranted.

### 5.5. Denervation and Neuromuscular Junction Degradation

The skeletal muscle acetylcholine receptor (AChR) accepts the acetylcholine ligand diffusing across the synaptic cleft after being exported via exocytosis from the motor end plate of the innervating motor neuron when an action potential is propagated. AChR has 5 different subunits: α, β, δ, ε, and γ. The complex consists of 2α, 2β, 1δ, and 1ε (in mature muscle cells) or γ (in embryonic or denervated myofibers). Chrng (acetylcholine receptor subunit gamma) is only expressed in mature skeletal muscle after denervation [41]—with the ε subunit returning long after denervation [42]. We found a *log2fc of 3.599*, equivalent to a 1211% increase, in *Chrng* in 6m–28m (*R = 0.46* with CFAB); but *Chrne* (epsilon subunit) was increased by only *log2fc 0.50*, indicating an 860% relative increase of Chrng versus Chrne, suggesting increased denervation flux in older mice. This provides further evidence that denervated muscle fibers in older animals tend to be less robustly reinnervated than in younger animals [43]. Eventually, myofiber death and muscle atrophy occurs in fibers that are not reinnervated [43]. Additionally, older animals may exhibit a fiber type shift to a more type 1 slow twitch fiber composition. This occurs as the denervated type 2 muscle fibers (generally larger and producing more force at a greater contractile velocity than type 1 myofibers) are more often reinnervated with type 1 motor neurons in older animals, which then drives a phenotypic shift towards the type 1 myofiber [44]. This combination of switching to less powerful myofiber type (power equaling force times velocity), coupled with an overall loss in the total number of fibers (not to mention atrophy, and thus loss of force, from other causes) may lead to a reduction in peak power generation in older muscle [11].

The formation of the motor endplate, in particular, the clustering of ACHRs is propagated by the release of agrin by a motor neuron, which binds to the MuSK receptor (and dystroclycan and laminin to form a stabile scaffold), causing MuSK to phosphorylate and to downstream activate and recruit casein kinase 2 (Csnk2), rapsyn (Rapsn), and Dok-7 to form the ACHR clusters. Motor neuron outgrowth and attachment to myofibers requires the expression of MuSK and Agrin at the motor end plate [45]. Agrin acts to stabilize the MuSK receptor to the extracellular matrix and the cytoskeleton, forming a focal point for ACHR clustering [46]. However, Musk and Agrin genes did not significantly alter in our samples (log2fc −0.627 and −0.390, respectively). On the other hand, DOK4 is a peptide involved in neuronal outgrowth that was significantly declined (gene log2fc −0.64), and is upstream of both Rap1 (a g-coupled protein) and the ERK pathway. Interestingly, Trim9 declined 5.69-fold in our 28m TA (log2fc −2.51). Trim9 is a negative regulator of synaptic vesicle transmission that acts as a ubiquitin ligase to regulate the SNARE complex formation, and is important for axon guidance [47,48]. These findings of our current study suggest that if the gene expression changes are truly linked to protein expression alterations and resulting mechanisms, then reinnervation may be failing due to failures in axon guidance and outgrowth, per se, not from failure to attach to the motor endplate. Note that the top gene ontology enrichments from GOrilla include G protein-coupled acetylcholine receptor activity (*enrichment 26.29*), axon guidance receptor activity (*enrichment 14.32*), and acetylcholine receptor activity (*enrichment 13.63*), all of which hint at alterations in gene expression related to the motor endplate (see Table S5). Obviously, more work is needed to confirm this hypothesis.

A recent RNAseq study of the TA muscle using a mouse stroke model in 5 m mice found altered transcriptomes compared to age-matched non-stroke mice including: an upregulation in stroke mice >log2fc1 of Gadd45a, Shroom3, Chrna2, and MuSK, along with downregulation (log2fc < −1) of P2ry1 [49]. Corresponding to the Ferrandi and colleagues findings, our study found some similar changes in 6m to 28m TA: *Chrna9 (log2fc1.05, padj = 6.01* × *10*^−*6*^*, R = 0.44 with CFAB); Gadd45a (log2fc 2.11, padj = 4.2* × *10*^−*11*^*, R = 0.57 with CFAB); and Gadd45g (log2fc 1.18, padj = 1.1* × *10*^−*5*^*, R = 0.73 with CFAB)*; Shroom3 was not significantly different, but *Shroom4 decreased (*−*1.94 log2fc, padj = 0.016*); as did *P2ry1 (log2fc* −*1.08, padj = 0.048, R = 0.765 with CFAB)*. This suggests that some age-associated denervation related transcriptomic changes in normal aging may resemble stroke-induced effects found in younger mice.

### 5.6. DEGs Analysis

Barns and colleagues used gene arrays on quadriceps and gastrocnemius muscles from n = 4 female mice at various age groups (3m, 15, 24, 29m) to compare 24m to 29m mice and found changes in neuromuscular junction genes, similar to our own observations in male TA [50]. In that study, the baseline group were juvenile 3-month (long bone growth does not cease until around 4.5m) female mice, which makes direct comparison to our study complicated, particularly since the groups compared were 3m–15m, 15m–24m, 15m–29m, and 24m–29m [50]. Schaum and colleagues in 2020 published a comprehensive NGS RNAseq study of the TA muscle across the lifespan [15]. This study had both female (n = 2) and male mice (n = 4), but did not examine any functional data, though they did compare gene expression to protein expression.

Shavlakadze and colleagues [16] completed a robust multiple tissue rat gene expression map (including gastrocnemius muscle) in 7 different age groups and noted 13 genes that changed with age in common in 4 tissues examined, including *Psmb8 (in our study: log2fc 1.47, R = 0.56 with CFAB)*, *Tspo (our study: log2fc1.6, R = 0.73 with CFAB)*, *Irf7 (in our study: log2fc 1.18, R = 0.56 with CFAB), Ms4aga (aka: Cda01, in our study: Cda log2fc 1.04, R = 0.44 with CFAB), Isg15 (in our study: log2fc 1.50, R = 0.57 with CFAB).* The Shavlakadze study promotes the hypothesis that there are common age-related gene expression changes across multiple tissues and potentially species. Therefore, a similar experiment using mice would be instructive to note any differences between rat and mouse age-related gene expression common over multiple tissues [16].

With the ubiquitous use of the mouse model in aging, mechanistic, and pharmaceutical research, understanding both parallels and differences in age-associated gene expression with humans as related to functional decline is a necessary future undertaking. A recent comparative study of gene array data of skeletal muscle in mice and humans by Zhuang and colleagues revealed 249 homologous overlapping age-related genes, but noted 6333 differentially expressed skeletal muscle genes between under 30-year-olds and over 65-year-old humans—very similar to our finding of *6587* in 6m–28m; however, the ages of the mice were not given in this study, so comparisons to our work are limited [17]. It is important to note that the age of the older mice plays a key role in differential gene expression, as we have uncovered in our study. More research is needed to establish age-associated gene expression changes related to functional decline in humans, and which of these overlap with mice.

### 5.7. Temporal Signatures in Gene Expression

According to our findings, mice experience a rapid transcriptomic change between 24m and 28m, suggesting that mice at the older age are experiencing far more age-related changes than relatively younger mice—in effect, an acceleration of age-related changes occurred in the 28m compared to the 24m. Kang and colleagues demonstrated a similar gene expression pattern to our observation, finding a greater change in 28m versus 24m mouse muscle [51]. They observed gene expression changes to be accelerating as mice age from 24m to 28m. They proposed that distinct aging profiles exist at early/gradual aging (24m) and late/accelerated aging (28m). However, their study used a juvenile reference group (aged 2m), making direct data comparisons to our current study difficult, because their control group were adolescent mice still experiencing long bone growth [51].

Recent work by Schaum and colleagues also underscores the importance of temporal relationships in RNAseq data [15]. Schaum and colleagues performed an elegant and comprehensive analysis of multiple tissues (17 organs) over multiple timepoints (10 ages), albeit with only n = 4 per group. They note that there are distinct patterns of aging conserved between organ systems that differ in their age of onset and degree of change. Of particular interest to us was their inclusion of tibialis anterior muscle as one of the representative organs. They show a vast increase in differential expression between 27m compared to 24m; similar to our findings of increased changes between 6m–24m and 6m–28m. However, direct comparisons of datasets between the studies are complicated by their use of 3m controls for differential gene expression and use of different cut-off criteria (see https://twc-stanford.shinyapps.io/maca/, accessed on 3 March 2022).

Interestingly, the PCA analysis of the DEGs (Figure 4) suggests distinct groupings of the older mice. For example, one group in the 24m completely overlaps with the 6m. In the 28m, there is one grouping closer to 6m and one further away. Speculatively, we suggest that those with PCA signatures closer to that of the younger mice may reflect mice undergoing so-called successful aging. However, more work is needed to determine these relationships, which may indicate different levels of gene expression based upon differential functional aging.

### 5.8. Bedrest, Disuse Atrophy, and Exercise

As reported by Mahmassani et al. 2019, after a 5-day period of bedrest, 61 genes were differentially expressed (pre-post) in the vastus lateralis of younger adults compared to older, with 51 of these genes changing only in young adults to levels equivalent to older adults at baseline, suggesting in some ways that older muscle resembles adult muscle suffering from disuse atrophy [52]. In our study we determined that of the top 10 genes they touted as being differentially upregulated in younger mice during bedrest, in our oldest mice *Fasn*, *Pfkfb3*, and *Rps4x* were significantly expressed differentially from adult mice (with *log2fc of* −*0.679,* −*1.09, and 0.93*, respectively). However, in their top 10 downregulated genes in adult humans after bedrest, only *Nov (*−*0.545 log2fc, trend padj = 0.067), Apln (0.81 log2fc, trend padj = 0.067),* and *Myl12a (1.39 log2fc, padj = 4* × *10*^−*10*^*)* were altered in our 28-month-old group compared to the 6-month. Fisher and colleagues used tetrodotoxin administration as a model of reversible denervation-induced disuse atrophy, and demonstrated that there was a time course dependent relationship for various gene expression changes [53]. With four of their top 7 differentially expressed atrophy-related genes also showing changes in our 28-month-old mice, if protein expression changes are similar to the gene expression changes, our data suggests that older muscle dysfunction may partly be due to chronic disuse patterns [53]. This presents an intriguing concept for future deliberation to determine which elements of acute detraining/disuse could be contributing to long-term disuse atrophy in older adults and which of these might be ablated by minimal increases in activity rates or other interventions to preserve function.

Exercise in its many forms has been shown to improve or preserve physical function during aging [3,10,18,54–56]. In recent work, Pillon and colleagues sought to catalogue gene expression changes resulting from exercise in human skeletal muscle using meta-analysis techniques on 66 previously published datasets [57]. Interestingly, they found 25 genes that changed commonly in both aerobic and resistance training. However, they included all genes with a false discovery rate (FDR) <0.1%, and had no cut-off standard for log2fc—making comparisons to our more strictly controlled dataset challenging. That being said, a number of the genes (or similar homologues in mice) identified as responsive to exercise and inactivity (Table 3 in Pillon et al.) were also found in our study to both be age-related and strongly associated with function (CFAB), including the following: *Cyr61 (log2fc 1.2, R* = −*0.83), Gadd45g (log2fc 1.2, R* = −*0.78), Ankrnd1 (log2fc 2.7, R* = −*0.75), Dyrk2 (log2fc* −*1.3, R = 0.73), Itga1 (log2fc* −*1.2, R = 0.84)* but not ITGA6 (Pillon et al.), *Atf7 (log2fc* −*1.6, R = 0.73)* but not ATF3 (Pillon et al.), Lgals3 *(log2fc 2.0, R = 0.78)* but not LGALSL (Pillon et al.,), *Nr1d1 (log2fc* −*1.0, R = 0.83)* and *Nr5a2 (log2fc* −*1.728, R = 0.77)* but not NR4A3 (Pillon et al.), FHL5 (log2fc −1.4, R = 0.72) but not FHL3 (Pillon et al.), two of the *COL family (Col4a4 and Col6a6)* but not COL3A1, COL4A4 or COL1A2 (Pillon et al.), and *Myh10* but not MYH1 or MYH9 (Pillon et al.). Using the authors’ elegant meta-analysis tool (found at https://metamex.eu/, accessed on 12 May 2023), we examined our top 71 DEGs (|R| > 0.80) with CFAB in human studies and found that, of those annotated by the authors, Plekhg1, Kdr, Pde4a, Pcdh12, Lynx1, Tspan18, Akap12, Col4a3, Psd3, Atp2b4, Heatr5a, Hspg2, Col4a2, Lama3, Slc25a36, Col4a1, Amy1, and Depdc7 were altered in expression by exercise in humans, while Cebpb was affected by inactivity but did not change with exercise. This data highlights the translatable importance of our gene set, while suggesting that exercise might alter the expression of many of these genes to potentially result in improved physical function.

### 5.9. Caveats

First of all, it is well-established that alterations in gene expression are often not equivalent to alterations in protein expression; in effect, the transcriptome ≠ proteome. We also fully acknowledge that correlation ≠ causation, and any potential links between DEGs and function need further mechanistic (i.e., gain/loss of function) determination to be validated. Thus, our upcoming work will investigate the protein abundance of physiologically relevant gene expression changes. Furthermore, we will begin to determine cellular signaling mechanisms connecting the numerous potential sequences of events.

Secondly, this study included male mice at three age-groups, and a single muscle, so any sexual dimorphisms or muscle-specific differences were not explored. We assumed that the TA muscle was representative of any of the primarily fast twitch ambulatory muscles throughout the mouse and, thus, that age-related gene expression changes would be similar in those other muscles. We used the entire TA to isolate total RNA to ensure all tissues of the muscle organ were represented. Future work examining single cell RNAseq and spatial RNAseq is a natural follow-up to the current study.

## 6. Conclusions

Despite the limitations noted above, we believe this dataset demonstrates novel potential links between age-associated changes in skeletal gene expression and overall physical function status. Importantly, we identified the differential gene expression of components and biological processes connected to the motor end plate as being both highly altered with aging and having strong associations with physical function. Additionally, we provided evidence that age-related changes and association with physical function of DEGs was accelerated in mice between 24m and 28m, hinting at a need for more investigation of mice at older ages. While more work is needed to determine the physiological relevance of the many changes uncovered, and to determine proteomic alterations and sexual dimorphisms, the multiple datasets presented herein may provide leads to help begin to characterize cellular mechanisms responsible for how age induces declines in muscle health and physical function.

## Supplementary Material

JAL3020013 Supplementary Material

## Figures and Tables

**Table 1.** Mouse Characteristics.

Age	n	Body Mass	Total Muscle	TA

months		g	mg	mg
6	8	33.04 ± 0.42	286.75 ± 6.97	58.39 ± 1.30
24	8	33.71 ± 0.67	254.52 ± 7.27 ^a^	50.14 ± 1.96 ^a^
28	8	31.01 ± 1.05	206.13 ± 5.75 ^b^	43.48 ± 0.96 ^b^

Body mass is the weight at tissue collection; Total Muscle is the combined mass of the mean extensor digitorum longus, tibialis anterior (TA), gastrocnemius, plantaris, and soleus muscle. Statistics are from a simple one-way ANOVA; different letters equal statistical significance at *p* < 0.05 using a Least Significant Differences post hoc test:

a different from 6m, and

b different from 6m and 24m.

**Table 2.** Top 30 Upregulated Aging Genes:

Gene_Id	AKA	NCIB Gene #	MGI #	log2fc	padj	Type

Bpifb1	LPLUNC1	228801	2137431	4.53	2.26 × 10^−3^	pc
Krt18	CK18, Endo B	16668	96692	4.49	1.43 × 10^−5^	pc
Ubd	Diubiquitin, FAT10	24108	1344410	4.46	1.38 × 10^−4^	pc
Sln	2310045A07Rik	66402	1913652	4.33	1.08 × 10^−6^	pc
Tac4	HK-1	93670	193,130	4.28	2.42 × 10^−3^	pc
Sprr1a	SPR1a	20753	106660	3.89	3.38 × 10^−3^	pc
Syt4	SytIV	20983	101759	3.85	2.65 × 10^−3^	pc
Dntt	Tdt	21673	98659	3.74	8.49 × 10^−4^	pc
Atp13a4	4631413J11Rik	224079	1924456	3.71	3.68 × 10^−3^	pc
Hamp2	HEPC2	66438	2153530	3.68	6.02 × 10^−2^	pc
1300002K09Rik	Stra6l, Rbpr2	74152	1921402	3.67	1.97 × 10^−2^	pc
4930558C23Rik	Ctxnd2	67654	1914904	3.66	7.45 × 10^−3^	pc
Ccl17	Scya17, TARC	20295	1329039	3.65	7.98 × 10^−3^	pc
1110059M19Rik	Prr32	68800	1916050	3.61	1.52 × 10^−5^	pc
Chrng	Achr-3, Acrg	11449	87895	3.60	1.78 × 10^−4^	pc
AA467197	NMES1	433470	3034182	3.59	2.21 × 10^−6^	pc
Neil3	C85903	234258	2384588	3.56	3.90 × 10^−3^	pc
Nppb	BNP, BNF	18158	97368	3.55	2.93 × 10^−3^	pc
Erc2	CAST, ELKS	238988	1098749	3.54	4.92 × 10^−20^	pc
Orm2	Orm-2, Agp1	18406	97444	3.53	9.31 × 10^−3^	pc
C130026I21Rik	4930565N07Rik	620078	3612702	3.51	8.89 × 10^−3^	pc
Olig1	Bhlhb6	50914	1355334	3.41	5.99 × 10^−3^	pc
F10	Cf10, Al1947	14058	103107	3.38	7.35 × 10^−4^	pc
Igfbp2	IBP-2	16008	96437	3.30	1.30 × 10^−2^	pc
Gbp1	Gbp2b, Mpa1	14468	95666	3.28	2.69 × 10^−2^	pc
Gm7609	EG665378	665378	3644536	3.27	2.67 × 10^−3^	pc
Gdf5	brp, CDMP-1	14563	95688	3.26	3.01 × 10^−11^	pc
Cd5l	AIM, Api6	11801	1334419	3.16	1.33 × 10^−1^	pc
Krt8	Card2, EndoA	16691	96705	3.13	1.17 × 10^−2^	pc
Cdca5	Sororin p35	67849	1915099	3.08	2.52 × 10^−2^	pc

6m vs. 28m: The 30 upregulated genes with the greatest |log2fc|. AKA = also known as, NCIB Gene is from https://www.ncbi.nlm.nih.gov/gene (accessed 3 January 2021), MGI = Mouse Genome Informatics from http://www.informatics.jax.org/marker (accessed 3 January 2021), log2fc = log base 2 fold change, adj. *p* = multiple comparison adjusted *p*-value.

**Table 3.** Top 30 Downregulated Aging Genes:

Gene_Id	AKA	NCIB Gene	MGI	log2fc	padj	Type

9130404H23Rik	Themis3	74556	1921806	−4.51	3.35 × 10^−5^	pc
5330417C22Rik	Elapor1	229722	1923930	−3.24	1.21 × 10^−2^	pc
Nlrp1c-ps	Nalp1c	627984	3582962	−3.11	1.56 × 10^−2^	pseudo
Oxct2a	Scot-t1	64059	1891061	−3.02	1.97 × 10^−2^	pc
1700001K23Rik		69319	1916569	−2.95	3.18 × 10^−2^	lncRNA
Kcng1	AW536275	241794	3616086	−2.78	1.21 × 10^−2^	pc
Gpr165	6530406P05Rik	76206	1923456	−2.59	4.81 × 10^−2^	pc
Fbxo48	A630050E13Rik	319701	2442569	−2.58	2.98 × 10^−3^	pc
1700071M16Rik		73504	1920754	−2.56	1.55 × 10^−5^	lncRNA
1700001O22Rik	1700113K14Rik	73598	1923631	−2.54	6.18 × 10^−6^	pc
Prap1	Upa	22264	893573	−2.51	4.80 × 10^−4^	pc
E130008D07Rik		545207	3584523	−2.51	3.02 × 10^−3^	lncRNA
Hrh4	H4R	225192	2429635	−2.51	5.20 × 10^−2^	pc
Trim9	mKIAA0282	94090	2137354	−2.51	4.82 × 10^−2^	pc
Zfp366	DC-SCRIPT	238803	2178429	−2.48	9.45 × 10^−6^	pc
Grem2	Prdc	23893	1344367	−2.45	1.06 × 10^−9^	pc
Rgag1	Rtl9, Mar9	209540	2685231	−2.44	4.84 × 10^−3^	pc
Duox2	LNOX2	214593	3036280	−2.44	3.50 × 10^−2^	pc
Nos1	bNOS, nNOS	18125	97360	−2.41	8.87 × 10^−4^	pc
4932411E22Rik	Ankfn1, nmf9	382543	2686021	−2.41	5.31 × 10^−2^	pc
Epha3	Cek4, End3	13837	99612	−2.38	2.15 × 10^−7^	pc
Il1rl2	IL-1Rrp2	107527	1913107	−2.35	4.27 × 10^−6^	pc
Nptxr	NPCD, NPR	73340	1920590	−2.34	2.83 × 10^−3^	pc
2700086A05Rik	Hoxaas3	72628	1919878	−2.31	1.53 × 10^−4^	anti-IncRNA
Gm16982		100036523	4439906	−2.28	6.93 × 10^−3^	IncRNA
Nrk	Nesk	27206	1351326	−2.27	3.87 × 10^−2^	pc
Hist1h2af	H2ac10, H2a-22	319173	2448309	−2.26	1.09 × 10^−2^	pc
Tll2		24087	1346044	−2.24	3.50 × 10^−2^	pc
Igsf9b	AI414108	235086	2685354	−2.21	3.63 × 10^−3^	pc
Necab1	Efcbp1, STIP-1	69352	1916602	−2.20	1.95 × 10^−3^	pc

6m vs. 28m: The 30 downregulated genes with the greatest |log2fc|. AKA = also known as, NCIB Gene is from https://www.ncbi.nlm.nih.gov/gene (accessed on 3 January 2021), MGI = Mouse Genome Informatics from http://www.informatics.jax.org/marker (accessed on 3 January 2021), log2fc = log base 2 fold change, adj. *p* = multiple comparison adjusted *p*-value.

**Table 4.** Age-Regulated Genes Associated with Physical Function:

Gene_Id	Slope	R	R^2^	Intercept	pval	log2fc	padj

Dclk3	0.041	0.899	0.809	3.864	5.073 × 10^−6^	−1.025	6.75 × 10^−4^
Plekhg1	0.085	0.896	0.803	7.093	6.124 × 10^−6^	−1.141	6.29 × 10^−11^
Zfp750	0.078	0.895	0.801	4.812	6.525 × 10^−6^	−1.571	1.12 × 10^−9^
Gabrd	−0.053	−0.886	0.784	3.839	1.125 × 10^−5^	1.201	4.12 × 10^−5^
Erc2	−0.172	−0.882	0.778	4.290	1.357 × 10^−5^	3.543	4.92 × 10^−20^
Ier3	−0.092	−0.881	0.777	7.172	1.405 × 10^−5^	1.227	3.14 × 10^−13^
P2ry1	0.086	0.881	0.776	8.163	1.456 × 10^−5^	−1.076	2.00 × 10^−10^
Kdr	0.114	0.880	0.775	10.616	1.506 × 10^−5^	−1.280	1.55 × 10^−8^
Pde4a	0.099	0.877	0.770	10.901	1.731 × 10^−5^	−1.211	3.34 × 10^−14^
Zyg11a	0.057	0.875	0.765	4.134	1.973 × 10^−5^	−1.329	1.51 × 10^−5^
Pcdh12	0.112	0.873	0.762	7.446	2.161 × 10^−5^	−1.273	2.35 × 10^−5^
Lynx1	0.096	0.873	0.762	12.112	2.162 × 10^−5^	−1.052	5.13 × 10^−7^
Lhfpl4	−0.047	−0.855	0.731	4.098	4.851 × 10^−5^	1.015	9.99 × 10^−5^
Tspan18	0.080	0.855	0.731	5.875	4.912 × 10^−5^	−1.120	4.08 × 10^−5^
Kcng4	0.104	0.853	0.728	10.729	5.212 × 10^−5^	−1.327	2.34 × 10^−16^
BC051142	−0.061	−0.850	0.723	2.516	5.983 × 10^−5^	2.462	2.77 × 10^−5^
Cacna2d4	0.118	0.849	0.721	8.663	6.176 × 10^−5^	−1.435	9.73 × 10^−9^
Spint2	−0.097	−0.844	0.712	6.437	7.667 × 10^−5^	1.436	3.42 × 10^−14^
Cyp1a1	−0.076	−0.842	0.709	4.882	8.145 × 10^−5^	1.211	1.04 × 10^−5^
Mmp15	0.088	0.841	0.707	9.482	8.653 × 10^−5^	−1.069	1.31 × 10^−8^
Vwa3a	0.066	0.841	0.707	4.803	8.676 × 10^−5^	−1.184	1.32 × 10^−4^
Frem1	0.055	0.839	0.704	4.218	9.120 × 10^−5^	−1.214	1.40 × 10^−4^
Gm5105	0.153	0.839	0.704	7.955	9.232 × 10^−5^	−1.930	2.69 × 10^−8^
Mfap3l	0.078	0.838	0.703	7.795	9.483 × 10^−5^	−1.089	2.27 × 10^−15^
Dnmt3a	0.090	0.837	0.701	10.929	9.949 × 10^−5^	−1.163	2.66 × 10^−15^
Rbm3	−0.104	−0.837	0.700	9.704	1.000 × 10^−4^	1.355	1.18 × 10^−12^
Akap12	0.082	0.836	0.698	8.347	1.050 × 10^−4^	−1.048	5.10 × 10^−9^
Col4a3	0.072	0.835	0.697	5.853	1.075 × 10^−4^	−1.081	7.96 × 10^−6^
Psd3	0.094	0.833	0.695	9.824	1.134 × 10^−4^	−1.119	6.34 × 10^−8^
Atp2b4	0.085	0.833	0.695	8.714	1.137 × 10^−4^	−1.134	5.74 × 10^−15^

6m vs. 28m (R^2^ ≥ 0.70) log2fc = log base 2 fold change, padj = multiple comparison adjusted *p*-value; R = correlation association, R^2^ = coefficient of determination; pval, slope and intercept from simple linear regression of the normalized log2fc of each gene from each mouse as the independent variable with the corresponding CFAB value as the dependent variable.

## Data Availability

The raw NGS RNAseq data is deposited in GEO (GSE152133). We have provided differential expression spreadsheets, additional figures/graphs, and methods in the Online Only Supplementary Files. Other data will be provided upon reasonable request to the corresponding author.
