# Supplementary material for: Skeletal Muscle Transcriptome Alterations Related to Declining Physical Function in Older Mice
Source: J Ageing Longev. Author manuscript; Available in PMC 2023 Oct 24. (PMC10597580; doi:10.3390/jal3020013)
Supplement: JAL3020013 Supplementary Material [file eFigure2 GOrilla 6m vs. 24m_vf.pdf › eFigure2 GOrilla 6m vs. 24m_vf.pdf]

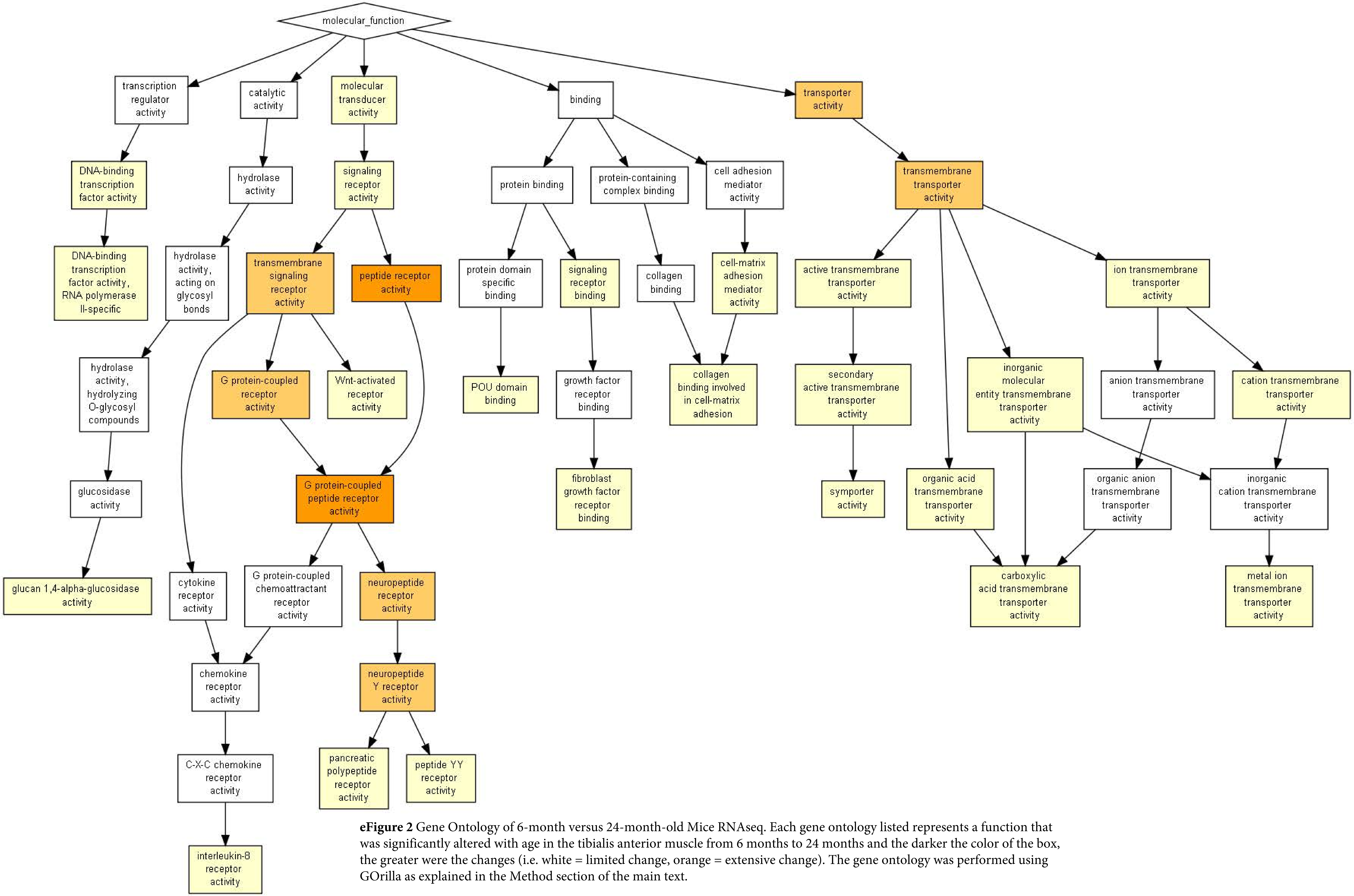

eFigure 2 Gene Ontology of 6-month versus 24-month-old Mice RNAseq. Each gene ontology listed represents a function that was significantly altered with age in the tibialis anterior muscle from 6 months to 24 months and the darker the color of the box, the greater were the changes (i.e. white = limited change, orange = extensive change). The gene ontology was performed using GOrilla as explained in the Method section of the main text.
