# Supplementary material for: Skeletal Muscle Transcriptome Alterations Related to Declining Physical Function in Older Mice
Source: J Ageing Longev. Author manuscript; Available in PMC 2023 Oct 24. (PMC10597580; doi:10.3390/jal3020013)
Supplement: JAL3020013 Supplementary Material [file NIHMS1905574-supplement-JAL3020013__Supplementary_Material.zip › eFigure1 GOrilla_6m vs. 28m_vf.pdf]

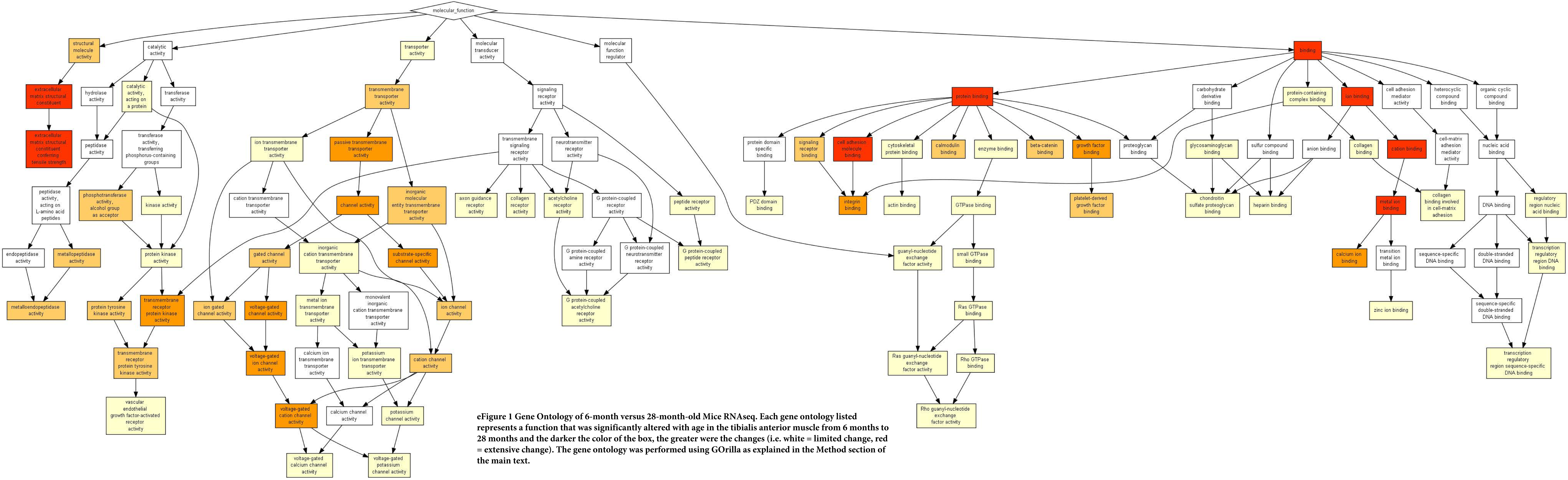

eFigure 1 Gene Ontology of 6-month versus 28-month-old Mice RNAseq. Each gene ontology listed represents a function that was significantly altered with age in the tibialis anterior muscle from 6 months to 28 months and the darker the color of the box, the greater were the changes (i.e. white = limited change, red = extensive change). The gene ontology was performed using GOrilla as explained in the Method section of the main text.
