# Supplementary material for: Skeletal Muscle Transcriptome Alterations Related to Declining Physical Function in Older Mice
Source: J Ageing Longev. Author manuscript; Available in PMC 2023 Oct 24. (PMC10597580; doi:10.3390/jal3020013)
Supplement: JAL3020013 Supplementary Material [file NIHMS1905574-supplement-JAL3020013__Supplementary_Material.zip › Graber et al._NGS_Online Supplemental Section_vf.pdf]

# Online Supplemental Section to:
Skeletal Muscle Transcriptome Alterations

Related to Declining Physical Function in Older Mice

Ted G. Graber, PhD[1,*], Rosario Maroto, MD PhD[2],
Jill Thompson, MS[2], Steve Widen, PhD[2], Zhaohui Man, MS[3],
Megan L. Pajski, PhD[1], Blake B. Rasmussen, PhD[2]

[1]Dept. of Physical Therapy, East Carolina University, [2]Dept. of Biochemistry & Molecular Biology, University of Texas Medical Branch, [3] Bioinformatics and Analytics Research Collaborative, University of North Carolina-Chapel Hill

*Corresponding Author:
Ted G. Graber, PhD
East Carolina University
Department of Physical Therapy
600 Moye Blvd.
Greenville, NC 27834
Email: grabert19@ecu.edu

Note: Zhaohui Man is currently at Duke University ( Durham, NC), and Megan L. Pajski is currently at ThermoScientific (Greenville, NC).

## Table of Contents:

| Title | Page |
|---|---|
| **eMethods** | **2-3** |
| **eResults** | |
| eTable S1 CFAB Determinants: Mean Standard Score | |
| **eDiscussion** | **4-5** |
| *Evidence for Potential Mechanisms* | |
| Disuse Atrophy | **4** |
| Calcium Handling Dysregulation | **4-5** |
| **eReferences** | **6** |
| **Other Supplemental Files** | |
| eFigure S1 GO Map 6m vs. 28m | PDF |
| eFigure S2 GO Map 6m vs. 24m | PDF |
| eTable S2-S3 Age-Related DEGs | Excel File |
| eTable S4-S9 GSEA and GOrilla | Excel File |
| eTable S10-S21 DEGs Strongly Associated with CFAB | Excel File |
| eTable S22-S23 DEGs Strongly Associated with TA Mass | Excel File |
| eTable S24 Enrich Results | Excel File |

## eMethods

### Functional Tests Comprising CFAB: See previous publications for further details.[1-5]

A) *Activity/Volitional Exercise Rate* (voluntary wheel running): To track activity, the mice were singly housed with a running wheel (Columbus Instruments) for 1 week. The number of revolutions of the wheel over the week were converted to km/day and reported as such.

B) *Rotarod:* To quantify overall neuromuscular function (endurance, power production, balance and coordination) we used a Panlab Rota-Rod. The procedure involved two training sessions, one per day (three trials per day, minimum of 15 minutes rest between trials), to acclimate the mice to the device, followed by a testing day in which three trials were performed, once again with minimum of 15-minute rest periods between trials. The maximum number of seconds the mouse remained on the rotarod before falling was the outcome measure.

C) *Grip Test Meter:* To directly measure grip strength of the mice, we used a Bioseb grip strength meter. Five trials for forelimb grip was performed in one session. For one trial, the mouse was removed from its cage, held by the tail and placed, gently, so that its paws can grab the bar/grid. Then the mouse was smoothly pulled backed until it releases the bar/grid. We report the highest of the five trials in Newtons as the outcome measure.

D) *Inverted Cling Grip Test:* This test was used to quantify muscle strength and endurance. The mouse gripped a grid (a custom-built device was used) and was inverted. The outcome is latency until falling, in seconds, to the padded surface below, best out of two trials (7 minute ceiling). The mice were evaluated two times on one day with a minimum of 15 minutes between trials for a rest period. If a mouse held on for less than 10 seconds it was be immediately retested to determine if the fall was a slip. If a slip occurred, that trial did not count towards the two maximum trials.

E) *Treadmill:* The mice were assessed for endurance, maximum running speed and aerobic capacity by running on a treadmill (Columbus Instruments 6 mouse treadmill). The outcome measurement is the length of time run in seconds. The mice were introduced to the device gradually with two acclimation trials over 2 days. Initially, in the first session the mice were introduced to the device and learned to walk (walking speed, 3 m/min, for two minutes maximum), then in the second session the first session was repeated, followed by a second session where the speed was accelerated as they learn to run (3 m/min, accelerated at 0.6 m/min/20sec; 2

minutes maximum) During actual testing (day 3) the mouse started at 3 m/min and the treadmill is accelerated at 0.6m/min/20sec until the mice reached exhaustion/failure, with the outcome measure as the number of seconds before failure. Failure was inability to keep up with the treadmill resulting in accumulation of 3 shocks from the shock grid (set at 3 mA).

| Test | 6m | 24m | 28m |
|---|---|---|---|
| **VWR** | **-0.730** | **-0.911** | **-1.196[a]** |
| **Grip Meter** | **0.039** | **-0.897[a]** | **-1.494[a,b]** |
| **Inverted Cling (log10)** | **0.491** | **-1.820[a]** | **-2.459[a]** |
| **Rotarod** | **-0.633** | **-1.060** | **-1.706[a,b]** |
| **Treadmill** | **-0.583** | **-0.237** | **-0.597** |

**eTable S1 Standardized scores for CFAB Determinants**. **NOTE:** This table has the data from the randomly selected subset of mice (total RNA isolated from the tibialis anterior muscle of each mouse for RNAseq) from the parent study[1] and does not equate to the original dataset which had a much larger n per group. **Key:** values = means of the standardized values for the individual mice used in this study based upon the overall 6m mean and standard deviation (previously reported[1]). m = months of age, VWR = voluntary wheel running, CFAB = comprehensive functional assessment battery, different letters indicate statistical difference ($p<0.05$ from 1-way ANOVA, LSD posthoc test): a = significantly different from 6m, b = significantly different from 24m

## Online Supplemental Discussion

### Further Potential Mechanisms of Age-Related Neuromuscular Decline

*(NOTE: data from our current study presented in italics)*

**Calcium Handling Dysregulation (Implications and Effects):** Increased sarcoplasmic calcium levels may alter numerous signaling pathways relying upon a $Ca^{+2}$ second messenger. In skeletal muscle the binding of $Ca^{+2}$ to troponinC, causes a conformation shift of the troponin complex moving tropomyosin to expose the myosin binding site on actin, allowing for cross-bridge cycling. Normally this process is induced by a calcium influx from the sarcoplasmic reticulum when the ryanodyne (RYR) receptor is prompted to open by the voltage-gated dihydropyridine receptor responding to a propagating action potential. Relaxation is induced when $Ca^{+2}$ disassociates from troponinC as the sarcoplasmic endoplasmic reticulum ATPase (SERCA) pumps $Ca^{+2}$ against the concentration gradient from the sarcoplasm back into the sarcoplasmic reticulum using ATP.

Sarcolipin (*Sln, increased log2fc 4.33, adj.* $p=1.1x10^{-6}$), phospholamban (*Pln, increased log2fc 0.663, adj. p=0.0003*) and myoregulin (*Mrn, aka 2310015B20Rik*, did not significantly alter—though the gene is not yet fully annotated) are regulatory elements of SERCA that block SERCA pumping activity while allowing ATPase activity to consume energy and generate heat.[6] Notably, Lin and colleagues (with RNAseq) also found that in 3-month versus 24-month old mouse rectus femoris SLN was significantly upregulated.[7]

SLN and PLN are additive in effect and can cause super-inhibition of SERCA pumping activity when co-expressed. There are other newly identified micropeptides involved SERCA regulation, including DWORF (increasing SERCA pumping) and the negative regulators endoregulin and another-regulin.[8] Increased expression of these regulatory genes may be adaptive strategies for increasing non-shivering thermogenesis to ward off body temperature dysregulation in older mammals and/or to improve energy balance in more sedentary individuals; but may have adverse consequences concerning muscle and physical function.[9-10] However, this current study demonstrated that *SLN* expression is not only greatly over-expressed in 28m mice (*>12-fold*) but is negatively correlated (*R=-0.55*) with *CFAB* functional scores. One mechanism by which this could occur is by increasing the time needed to relax muscle fibers between contractions, by delaying disassociation of $Ca^{+2}$ from troponin due to an increased sarcoplasmic calcium concentration, potentially leading to decreased power production, albeit with a potential for improved fatigue resistance. Sarcolipin is overexpressed in Duchenne muscular dystrophy (DMD) patients and DMD transgenic mouse models, and the knockdown of SLN restores muscle and physical function.[11] However, knock-out of SLN prevents normal hypertrophic and fiber-type shift response to overloading, and increases ½relaxation rate compared to wild type.[12-13] Transgenic mice over-expressing SLN have been shown to have an increased metabolic rate, and ½ relaxation time, while increasing SLN in rat muscle has been shown to decrease both maximal isometric force and ½ relaxation time.[14-15] Thus the jury is still out on what effect SLN upregulation might have in older muscle.

## Online Only Supplemental Section

In addition to dysregulating cross-bridge cycling and force generation, overexpression of SLN and PLN leading to increased prevalence of cytosolic $Ca^{+2}$ abundance may stimulate numerous calcium-dependent signaling pathways. For example, increased levels of sarcoplasmic calcium can decrease promoter activity for CGRP (calcitonin gene-related peptide), which is alternatively spliced from the calcitonin gene. CGRP binds to the calcitonin receptor like receptor (CKACRL) which consists of three different subunits: the receptor component protein (Rcp), the calcitonin like receptor (*Calcl, log2fc-0.34, adj. p=0.006*), and the receptor activity-modifying protein 1 (Ramp1). *Ramp1 (log2fc 0.53, adj. p=0.01)* is involved in angiogenesis and wound healing. Thus, our data suggests potential downstream adverse effects related to alterations in $Ca^{+2}$ signaling.

## References:

References

1. Graber TG, Maroto R, Fry CS, Brightwell CR, Rasmussen BB. Measuring Exercise Capacity and Physical Function in Adult and Older Mice [published online ahead of print, 2020 Aug 21]. J Gerontol A Biol Sci Med Sci. 2021 Apr 30;76(5):819-824. doi: 10.1093/gerona/glaa205
2. Graber TG, Fry CS, Brightwell CR, Moro T, Maroto R, Bhattarai N, Porter C, Wakamiya M, Rasmussen BB. (2019). Skeletal Muscle Specific Knockout of DEP domain-containing 5 Increases mTORC1 Signaling, Muscle Cell Hypertrophy, and Mitochondrial Respiration. J Biol Chem. 2019;294(11):4091-4102. doi: 10.1074/jbc.RA118.005970. Epub 2019 Jan 11
3. Graber TG*, Ferguson-Stegall L*, Liu H, Thompson LV. (2015). Voluntary Aerobic Exercise Reverses Frailty in Old Mice. The Journals of Gerontology. Series A, Biological Sciences. 70(9): 1045-1058. doi: https://doi.org/10.1093/gerona/glu163 Epub ahead of print: 09/30/14.
4. Graber TG, Ferguson-Stegall L, Kim JH, Thompson LV. C57BL/6 neuromuscular healthspan scoring system. J Gerontol A Biol Sci Med Sci. 2013;68(11):1326-1336. doi:10.1093/gerona/glt032
5. Graber TG, Fry CS, Brightwell CR, et al. Skeletal muscle-specific knockout of DEP domain containing 5 protein increases mTORC1 signaling, muscle cell hypertrophy, and mitochondrial respiration. J Biol Chem. 2019;294(11):4091-4102.
6. Anderson DM, Anderson KM, Chang CL, et al. A micropeptide encoded by a putative long noncoding RNA regulates muscle performance. Cell. 2015;160(4):595-606. doi:10.1016/j.cell.2015.01.009
7. Lin IH, Chang JL, Hua K, Huang WC, Hsu MT, Chen YF. Skeletal muscle in aged mice reveals extensive transformation of muscle gene expression. BMC Genet. 2018 Aug 8;19(1):55. doi: 10.1186/s12863-018-0660-5. PMID: 30089464; PMCID: PMC6083496
8. Anderson DM, Makarewich CA, Anderson KM, et al. Widespread control of calcium signaling by a family of SERCA-inhibiting micropeptides. Sci Signal. 2016;9(457):ra119. Published 2016 Dec 6. doi:10.1126/scisignal.aaj1460
9. Soparila DH, Pant M, ShaikH SWA, et al. Sarcolipin overexpression improves muscle energetics and reduces fatigue. J Appl Physiol. 2015;118:1050–1058.doi: 10.1152/japplphysiol.01066.2014
10. Bal N, Maurya S, Sopariwala D, et al. Sarcolipin is a newly identified regulator of muscle-based thermogenesis in mammals. Nat Med. 2012;18:1575–1579. doi:10.1038/nm.2897
11. Voit A, Patel V, Pachon R, et al. Reducing sarcolipin expression mitigates Duchenne muscular dystrophy and associated cardiomyopathy in mice. Nat Commun. 2017;8(1):1068. Published 2017 Oct 20. doi:10.1038/s41467-017-01146-7
12. Tupling AR, Bombardier E, Gupta SC, et al. Enhanced Ca2+ transport and muscle relaxation in skeletal muscle from sarcolipin-null mice. Am J Physiol Cell Physiol. 2011;301(4):C841-C849. doi:10.1152/ajpcell.00409.2010
13. Fajardo VA, Rietze BA, Chambers PJ, et al. Effects of sarcolipin deletion on skeletal muscle adaptive responses to functional overload and unload. Am J Physiol Cell Physiol. 2017;313(2):C154-C161. doi:10.1152/ajpcell.00291.2016

14. Maurya SK, Bal NC, Sopariwala DH, et al. Sarcolipin Is a Key Determinant of the Basal Metabolic Rate, and Its Overexpression Enhances Energy Expenditure and Resistance against Diet-induced Obesity. J Biol Chem. 2015;290(17):10840-10849. doi:10.1074/jbc.M115.636878
15. Tupling AR, Asahi M, MacLennan DH. Sarcolipin overexpression in rat slow twitch muscle inhibits sarcoplasmic reticulum Ca2+ uptake and impairs contractile function. J Biol Chem. 2002;277(47):44740-44746. doi:10.1074/jbc.M206171200
